# Two-stage case-control association study of dopamine-related genes and migraine

**DOI:** 10.1186/1471-2350-10-95

**Published:** 2009-09-21

**Authors:** Roser Corominas, Marta Ribases, Montserrat Camiña, Ester Cuenca-León, Julio Pardo, Susana Boronat, María-Jesús Sobrido, Bru Cormand, Alfons Macaya

**Affiliations:** 1 Grup de Recerca en Neurologia Infantil, Hospital Universitari Vall d'Hebron, Barcelona, Spain; 2 Department of Psychiatry, Hospital Universitari Vall d'Hebron, Barcelona, Spain; 3 Fundación Pública Galega de Medicina Xenómica, Santiago de Compostela, Spain; 4 Servicio de Neurología, Hospital Clínico Universitario de Santiago de Compostela, Spain; 5 CIBER Enfermedades Raras, Instituto de Salud Carlos III, Spain; 6 Departament de Genètica, Facultat de Biologia, Universitat de Barcelona, Spain; 7 Institut de Biomedicina de la Universitat de Barcelona (IBUB), Spain

## Abstract

**Background:**

We previously reported risk haplotypes for two genes related with serotonin and dopamine metabolism: *MAOA *in migraine without aura and *DDC *in migraine with aura. Herein we investigate the contribution to migraine susceptibility of eight additional genes involved in dopamine neurotransmission.

**Methods:**

We performed a two-stage case-control association study of 50 tag single nucleotide polymorphisms (SNPs), selected according to genetic coverage parameters. The first analysis consisted of 263 patients and 274 controls and the replication study was composed by 259 cases and 287 controls. All cases were diagnosed according to ICHD-II criteria, were Spanish Caucasian, and were sex-matched with control subjects.

**Results:**

Single-marker analysis of the first population identified nominal associations of five genes with migraine. After applying a false discovery rate correction of 10%, the differences remained significant only for *DRD2 *(rs2283265) and *TH *(rs2070762). Multiple-marker analysis identified a five-marker T-C-G-C-G (rs12363125-rs2283265-rs2242592-rs1554929-rs2234689) risk haplotype in *DRD2 *and a two-marker A-C (rs6356-rs2070762) risk haplotype in *TH *that remained significant after correction by permutations. These results, however, were not replicated in the second independent cohort.

**Conclusion:**

The present study does not support the involvement of the *DRD1*, *DRD2*, *DRD3*, *DRD5*, *DBH*, *COMT*, *SLC6A3 *and *TH *genes in the genetic predisposition to migraine in the Spanish population.

## Background

Migraine is a highly prevalent neurological disorder involving multiple susceptibility genes and environmental factors [1,2]. The current clinical classification follows the International Criteria for Headache Disorders (ICHD-II), with the two main categories of migraine without aura (MO) and migraine with aura (MA) [3]. The pathophysiology of migraine is not entirely understood, but a role for dopamine (DA) was already suggested thirty years ago [4]. The DA hypothesis relies on the observed signs of central DA hypersensitivity in migraine patients and the known capacity of DA receptors to regulate nociception, vascular tone and autonomic responses [5]. Studies in animal models revealed that DA receptors are present in the trigeminovascular pathway and showed that DA can act as an inhibitor of nociceptive trigeminovascular transmission in the rat brain [6]. Along this line, DA antagonists have proved useful in aborting migraine headache or associated symptoms [5]. However, DA antagonists are not always selective and may act through DA receptor-independent mechanisms [7]. Also, a review of pharmacological and therapeutic studies in migraine could not provide convincing evidence of a direct role of DA in migraine pathogenesis [8].

Several association studies in different populations have focused on genes encoding proteins of the dopaminergic neurotransmission system, including DA receptors, the DA transporter, and enzymes involved in the synthesis and catabolism of DA. These studies provided conflicting results [7], although a recent, most comprehensive analysis of 10 dopamine-related genes in MA suggested that *DBH *and *SLC6A3*, at least, might be involved in migraine pathogenesis [9].

In a previous study that evaluated the contribution of 19 serotonin-related genes to migraine susceptibility in our cohort of Spanish migraineurs, we reported risk haplotypes in *MAOA *for migraine without aura and in *DDC *for migraine with aura [10], both genes being key players in the serotonin and dopamine metabolic pathways. In order to further elucidate the involvement of the dopaminergic system in migraine liability, nine dopamine-related genes were selected for a two-stage case-control association study in the Spanish population.

## Methods

### Subjects

Our initial sample (population 1) was recruited between 2002 and 2006 in Spain and consisted of 271 migraineurs (mean age 37 +/- 16 years) and 285 unrelated migraine-free controls (mean age 55 +/- 18) matched for ethnicity (Caucasian Spanish) and sex frequency (76% women). The follow-up replication study (population 2) consisted of 272 patients and 302 healthy controls recruited subsequently between 2006 and 2007, also in Spain. All patients were diagnosed by clinical neurologists in the team (M.J.S., B.N., S.B. or A.M.) as having MO (55.9% in population 1 and 61.4% in population 2) or MA based on the International Criteria for Headache Disorders 2^nd ^edition (ICHD-II) [3] after administration of a structured questionnaire and direct interview and examination. Patients were recruited from three centers (Hospital Universitari Vall d'Hebron, HUVH, Barcelona; Hospital Sant Joan de Déu, Manresa; Fundación Pública Galega de Medicina Xenómica, FPGMX, Santiago de Compostela). Patients with hemiplegic migraine, a MA variant usually showing monogenic inheritance, were excluded. The control samples consisted of Caucasian Spanish unrelated adult subjects (blood donors, individuals that underwent surgery unrelated to migraine or unaffected partners of migraine patients) that were matched for sex with patients and recruited in the same geographic areas (HUVH and FPGMX). Migraine and positive family history of migraine or any type of severe or recurrent headache in first - degree relatives were excluded in all control subjects through personal interview. Genomic DNA was isolated from peripheral leukocytes or saliva. Research was approved by the local Ethics Committees and all the adult participants, and the children or their parents gave their informed consent prior to participate in the study, according to the Helsinki Declaration.

### SNP selection, SNPlex design, genotyping and quality control

We selected SNPs in nine candidate genes involved in dopaminergic neurotransmission. These genes encode five DA receptors (*DRD1*, *DRD2*, *DRD3*, *DRD4 *and *DRD5*), three enzymes involved in DA degradation (*COMT *and *DBH*) or synthesis (*TH*), and the DA transporter (*SLC6A3 *or *DAT1*; [see Additional File 1]).

In order to ensure a full genetic coverage of these genes and to minimize redundancy, we used the LD-select software [11] and the HapMap database (; release 20)[12] to evaluate the LD pattern of the region spanning each candidate gene plus three to five kb flanking regions. TagSNPs were selected at an r^2 ^threshold of 0.85 and minimal allele frequency (MAF)>0.15 for genes with less than 15 tagSNPs and MAF>0.25 for those genes with 15 or more tagSNPs (*COMT*, *DBH *and *SLC6A3*). A total of 69 tagSNPs (26 in multi-loci bins and 43 singletons) were chosen [see Additional File 1]. Of these, five did not pass through the SNPlex design pipeline. After genotyping, MAF were determined in our control population 1 [see Additional File 2]. To ensure that no population stratification was present in the sample, 45 anonymous unlinked SNPs located at least 100 kb distant from known genes were also analyzed [13] by means of STRUCTURE [14], FSTAT [15] and the method by Pritchard and Rosenberg [16], as previously described [17].

Genotyping was performed at the Barcelona node of the National Genotyping Center  using the SNPlex technology [18]. Two CEPH DNA samples were included in the different genotyping assays, and a concordance rate of 100% with HapMap data was obtained. The allelic variants of the SNPs under study were named on the coding strand of each gene.

### Statistical analyses

The minimal statistical power, calculated *post hoc *in population 1 using the Genetic Power Calculator software [19], assuming a disease prevalence of 0.12, an odds ratio (OR) of 1.7, a significance level (α) of 0.05 and a MAF of 0.123, the lowest in control population, was 85% and decreased to 74% for MO and 68% for MA. Given these estimates, we decided to begin our study by performing a joint analysis of the MO and MA groups, and to proceed to separate analyses of clinical subgroups only if a positive association was obtained in the whole sample.

Individuals with <40% successful genotypes were excluded from the analysis; SNPs with >10% missing genotypes were considered as failed; SNPs at r^2 ^> 0.85 from any other studied SNP or showing deviation from Hardy-Weinberg equilibrium (HWE; threshold set at 0.01) as calculated in our control population 1 were also excluded. The SNPs analyzed in the follow-up population were also in HWE.

### Single-marker analysis

The analysis of HWE as well as case-control comparisons of both allele and genotype frequencies under a codominant model were performed with the SNPassoc R library [20] initially in population 1, adjusting by sex. When a nominal association was identified (P < 0.05), dominant and recessive models were also analyzed. The significance threshold under the Bonferroni correction for multiple testing was set at P < 5e-04 upon consideration of 50 SNPs analyzed, genotype and allele comparisons and a single clinical group. Under a False Discovery Rate (FDR) of 10% the threshold was set at P < 0.0035, using the qvalue R library [21].

### Multiple-marker analysis

Risk haplotypes were assessed in the whole migraine group (MO + MA) with the UNPHASED software [22], only for genes showing association in the single-marker analysis after the Bonferroni or FDR corrections. The best up to five-marker haplotype was selected as previously described [17]. Significance was estimated by a 10,000 permutation procedure with UNPHASED [22]. The specific assignment of haplotypes to individuals was performed independently in cases and controls with the PHASE 2.0 software [23]. The comparisons of risk haplotype carriers *vs *non carriers were performed using the SNPassoc R library [20] adjusting by sex. Subsequently, risk haplotypes originally identified in the all-migraine group and SNPs trespassing the FDR threshold were tested in the MO and MA subgroups.

### Follow-up replication study

Risk haplotypes identified in population 1 were tested in the replication population 2. For SNPs showing nominal association with migraine in population 1 a comparison of genotype and allele frequencies was undertaken in population 2.

## Results

Initially, 64 SNPs from nine candidate genes encoding proteins related with DA neurotransmission were genotyped [see Additional File 1]. Fourteen SNPs were excluded from statistical analysis after data depuration in the first population: ten had genotype call rates <90%, one was monomorphic and three were in strong LD with other SNPs (r^2 ^> 0.85, [see Additional File 1]). The 50 remaining SNPs had MAF>0.12 and were in HWE in control population 1 (P > 0.01; [see Additional File 2]). After the exclusion of individuals with low genotyping rate, population 1 consisted of 263 patients and 274 controls, and population 2 was composed of 259 patients and 287 controls. No evidence of population stratification was found in any of the two populations studied by applying the STRUCTURE software [see Additional File 3], the Fst coefficient (theta = 0, 99%CI = 0.000-0.001 for population 1 and theta = 0, 99%CI = 0.000-0.002 for population 2) and the method by Pritchard and Rosenberg (P = 0.57 for population 1 and P = 0.05 for population 2).

In the single-marker analysis, genotype and allele frequencies were compared between patients and controls in population 1 [see Additional File 2]. Six SNPs within five genes (*DRD1*, *DRD2*, *DRD3*, *DBH *and *TH*) displayed P-values < 0.05 (table 1). Two of them, rs2283265 in *DRD2 *and rs2070762 in *TH*, remained significant after applying a FDR of 10% (Table 1A) and were further considered for the multiple-marker analysis. No SNP withstood the restrictive Bonferroni correction for multiple testing. For these two SNPs we sought to detect a specific association with either one of the clinical subtypes, MO or MA. We found that in population 1, *DRD2 *rs2283265 was associated wit both MO and MA, while *TH *rs2070762 was only associated with MO (Table 1B).

**Table 1:** Results of dopamine-related genes association studies

**A. Significant results of the association study of 50 SNPs from eight dopamine-related genes in population 1 (patients N = 263, controls N = 274) and results of the analysis of these markers in population 2 (patients N = 259, controls N = 287)**.																
		**Cases**				**Controls**					**Genotype** **11 vs 12+22**		**Genotype 11+12 vs 22**		**Allele 2 vs allele 1**	
												
***Gene***	**SNP population**	**11**	**12**	**22**	**Sum**	**11**	**12**	**22**	**Sum**	**P value**	**OR (95% IC)**	**P value**	**OR (95% IC)**	**P value**	**OR (95% IC)**	**P value**

***DRD1***	rs251937 p1	133 (54.7)	95 (39.1)	15 (6.2)	243	120 (45.5)	119 (45.1)	25 (9.4)	264	NS	1.45^a ^(1.02-2.08)	0.0355	1.58^a ^(0.82-3.12)	NS	1.36 (1.04-1.79)	0.0261
	p2	130 (53.0)	95 (38.8)	20 (8.2)	245	144 (51.8)	116 (41.7)	18 (6.5)	278	NS	1.05^a ^(0.74-1.49)	NS	1.29 (0.66-2.51)	NS	1.01^a ^(0.77-1.33)	NS

***DRD2***	rs12363125 p1	133 (51.8)	97 (37.7)	27 (10.5)	257	108 (40.6)	128 (48.1)	30 (11.3)	266	0.0304	1.56^a ^(1.11-2.22)	0.0102	1.07^a ^(0.62-1.85)	NS	1.31 (1.01-1.70)	0.0401
	p2	112 (43.6)	123 (47.9)	22 (8.5)	257	141 (49.5)	112 (39.3)	32 (11.2)	285	NS	1.27 (0.90-1.78)	NS	1.35^a ^(0.76-2.38)	NS	1.07 (0.83-1.39)	NS

***DRD2***	rs2283265 p1	210 (82.4)	44 (17.3)	1 (0.3)	255	196 (72.3)	69 (25.5)	6 (2.2)	271	0.0085	1.79^a ^(1.18-2.70)	0.0059	5.88^a ^(0.68-50)	NS	1.78 (1.20-2.63)	0.0030*
	p2	199 (77.7)	54 (21.1)	3 (1.2)	256	226 (81.3)	49 (17.6)	3 (1.1)	278	NS	1.24 (0.81-1.89)	NS	1.09 (0.22-4.46)	NS	1.2^a ^(0.81-1.77)	NS

***DRD3***	rs3732790 p1	113 (44.0)	119 (46.3)	25 (9.7)	257	104 (38.7)	115 (42.7)	50 (18.6)	269	0.0131	1.25^a ^(0.88-1.75)	NS	2.12^a ^(1.27-3.57)	0.0033	1.36 (1.06-1.75)	0.0169
	p2	101 (39.5)	122 (47.7)	33 (12.8)	256	114 (40.3)	125 (44.2)	44 (15.5)	283	NS	1.03 (0.73-1.46)	NS	1.25^a ^(0.76-2.04)	NS	1.04 (0.81-1.33)	NS

***DBH***	rs1611131 p1	129 (56.6)	76 (33.3)	23 (10.1)	228	129 (49.6)	114 (43.8)	17 (6.6)	260	0.0400	1.33^a ^(0.93-1.92)	NS	1.57 (0.81-3.02)	NS	1.09 (0.83-1.47)	NS
	p2	124 (51.0)	102 (42.0)	17 (7.0)	243	136 (52.7)	107 (41.5)	15 (5.8)	258	NS	1.07 (0.76-1.52)	NS	1.22 (0.60-2.50)	NS	1.08^a ^(0.82-1.42)	NS

***TH***	rs2070762 p1	51 (20.8)	138 (56.3)	56 (22.9)	245	84 (32.2)	129 (49.4)	48 (18.4)	261	0.0130	1.81 (1.21-2.72)	0.0035*	1.32 (0.85-2.03)	NS	1.37^a ^(1.08-1.75)	0.0111
	p2	57 (23.4)	119 (49.0)	67 (27.6)	243	78 (28.2)	131 (47.3)	68 (24.5)	277	NS	1.28 (0.86-1.90)	NS	1.17 (0.79-1.74)	NS	1.16^a ^(0.92-1.49)	NS

**B. Results of the association study of *DRD2 *rs2283265 and *TH *rs2070762 in the migraine without aura and migraine with aura subgroups of population 1 and population 2**.																

		**Cases**				**Controls**					**Genotype** **11 vs 12+22**		**Genotype 11+12 vs 22**		**Allele 2 vs allele 1**	

***Gene***	**SNP population**	**11**	**12**	**22**	**Sum**	**11**	**12**	**22**	**Sum**	**P value**	**OR (95% IC)**	**P value**	**OR (95% IC)**	**P value**	**OR (95% IC)**	**P value**

**Migraine without aura**																

***DRD2***	rs2283265 p1	119 (82.6)	25 (17.4)	0 (0.0)	144	196 (72.3)	69 (25.5)	6 (2.2)	271	0.0097	1.81^a ^(1.10-3.03)	0.017	1.02 (1.004-1.04)	0.023	1.85 (1.15-2.90)	0.0081

	p2	115 (72.8)	40 (25.3)	3 (1.9)	158	226 (81.3)	49 (17.6)	3 (1.1)	278	NS	1.59 (1.00-2.53)	NS	1.80 (0.36-9.05)	NS	1.52^a ^(1.00-2.32)	NS

***TH***	rs2070762 p1	27 (19.0)	84 (59.2)	31 (21.8)	142	84 (32.2)	129 (49.4)	48 (18.4)	261	0.0143	2.04 (1.24-3.34)	0.0036	1.24 (0.74-2.05)	NS	1.40^a ^(1.04-1.88)	0.023

	p2	37 (24.5)	73 (48.3)	41 (27.2)	151	78 (28.2)	131 (47.3)	68 (24.5)	277	NS	1.19 (0.76-1.88)	NS	1.11 (0.71-1.76)	NS	1.11^a ^(0.84-1.47)	NS

**Migraine with aura**																

***DRD2***	rs2283265 p1	91 (82.0)	19 (17.1)	1 (0.9)	111	196 (72.3)	69 (25.5)	6 (2.2)	271	NS	1.75^a ^(1.00-3.03)	0.042	2.5^a ^(0.29-20.0)	NS	1.69 (1.01-2.77)	0.037

	p2	84 (85.7)	14 (14.3)	0 (0.0)	98	226 (81.3)	49 (17.6)	3 (1.1)	278	NS	1.37^a ^(0.72-2.56)	NS	1.01^a ^(0.99-1.02)	NS	1.41 (0.77-2.63)	NS

***TH***	rs2070762 p1	24 (23.3)	54 (52.4)	25 (24.3)	103	84 (32.2)	129 (49.4)	48 (18.4)	261	NS	1.57 (0.93-2.66)	NS	1.44 (0.83-2.5)	NS	1.35^a ^(0.98-1.89)	NS

	p2	20 (21.7)	46 (50.0)	26 (28.3)	92	78 (28.2)	131 (47.3)	68 (24.5)	277	NS	1.44 (0.82-2.52)	NS	1.28 (0.75-2.20)	NS	1.26^a ^(0.90-1.75)	NS

Abbreviations: CI, confidence interval; OR, odds ratio; SNP, single nucleotide polymorphism; NS, no significant; p1: population 1; p2: population 2

^a ^When odds ratio <1, the inverted score is shown.

* Statistically significant P-values after applying a false discovery rate of 10% (p < 0.0035)

rs251937: 1 = A, 2 = G; rs12363125: 1 = A, 2 = G; rs2283265: 1 = G, 2 = T; rs3732790: 1 = A, 2 = G; rs1611131: 1 = T, 2 = C; rs2070762: 1 = T, 2 = C.

The analysis of all possible allelic combinations within the *DRD2 *gene revealed a five-marker haplotype (rs12363125-rs2283265-rs2242592-rs1554929-rs2234689) associated with migraine (best adjusted P-value = 0.00889; Table 2), with an over-representation of the T-C-G-C-G allelic combination in cases (OR = 1.85, 95%CI = 1.13-3.04, P = 0.0139) and the C-A-A-C-C haplotype in controls (OR = 1.88, 95%CI = 1.25-2.82, P = 0.00199; Table 3). The T-C-G-C-G haplotype carriers displayed an OR of 1.74 (95%CI = 1.06-2.88, P = 0.0277). In order to investigate if the association was specific of MO or MA, we compared the risk haplotype carrier frequencies between controls and each migraine subgroup separately. Although the frequencies were different (10.6% controls, 17.7% MO, 16.4% MA), they only reached borderline significance in MO (P = 0.042), while no significant differences were found in MA (P = 0.12). We performed a second-stage replication study in an independent Spanish case-control cohort to test these positive findings. The frequency of the risk haplotype T-C-G-C-G (rs12363125-rs2283265-rs2242592-rs1554929-rs2234689) carriers in population 2 was compared between 259 patients and 287 controls. Control carrier frequencies were very similar to those obtained in population 1 (10.4% control population 2 and 10.8% control population 1), whereas case carriers were more frequent in population 1 (17.1%) than in population 2 (14.3%). Thereby, the differences between cases and controls in population 2 did not reach significance (P = 0.22).

**Table 2:** Haplotype analysis of *DRD2 *and *TH *SNPs in 263 migraine patients and 274 unrelated non-migraine controls using the UNPHASED software.

**Gene**	**Marker^*a *^Haplotype**	**Global P**	**Best Haplotype-P (Adjusted P-Value)**	**Risk Haplotype-OR**
***DRD2***	6 7	0.00519	0.00178 (0.00450)	1.37 (1.05-1.79)
	6 7 8	0.00175	9.74e-04 (0.00400)	1.74 (1.08-2.80)
	6 7 8 10	0.00135	0.00100 (0.00460)	1.84 (1.12-3.02)
	6 7 8 9 10^b^	0.00276	0.00199 (0.00889)	1.85 (1.13-3.04)

***TH***	1 2	0.0283	0.00573 (0.0150)	1.34 (0.94-1.90)

^*a *^***DRD2***: 6- rs12363125; 7- rs2283265; 8- rs2242592; 9- rs1554929; 10- rs2234689; ***TH***: 1- rs6356; 2-rs2070762.

^*b *^Best allelic combination (higher OR)

**Table 3:** Haplotype distributions of *DRD2 *in populations 1 and 2 using the UNPHASED software.

***DRD2***						
	**Population 1 Overall P-value = 0.00276**			**Population 2 Overall P-value = 0.048**		
		
**Marker^*a *^Haplotype**	**Cases (%)**	**Controls (%)**	**Haplotype-specific P; OR (CI)**	**Cases (%)**	**Controls (%)**	**Haplotype-specific P**
		
**6 7 8 9 10**						
C C G C G	49 (9.9)	58 (11.5)	0.423	66 (13.9)	47 (9.1)	0.018
C C G C C	47 (10.1)	40 (7.9)	0.233	42 (8.8)	55 (10.7)	0.331
C A A C C	40 (8.6)	76 (15.1)	0.00199; 1.88 (1.25-2.82) ^*c*^	53 (11.1)	53 (10.3)	0.660
T C G C G	44 (9.4)	27 (5.4)	0.0139; 1.85 (1.13-3.04)	37 (7.8)	28 (5.4)	0.136
T C A T C	287 (62.0)	303 (60.1)	0.581	278 (58.4)	333 (64.5)	0.047

***TH***						

	**Population 1 Overall P-value = 0.0283**			**Population 2 Overall P-value = 0.2**		
		
**Marker^*b *^Haplotype**	**Cases (%)**	**Controls (%)**	**Haplotype-specific P; OR (CI)**	**Cases (%)**	**Controls (%)**	**Haplotype-specific P**
		
**1 2**						
A C	82 (17.4)	68 (13.5)	0.0370; 1.34 (0.94-1.90)	100 (20.9)	86 (15.7)	0.129
A T	108 (23.0)	118 (23.7)	0.674	82 (17.2)	123 (22.4)	0.141
G C	162 (34.6)	149 (29.9)	0.127	147 (30.8)	178 (32.5)	0.935
G T	118 (25.0)	165 (32.9)	0.00573; 1.47 (1.11-1.94) ^*c*^	149 (31.1)	161 (29.4)	0.953

^*a *^***DRD2***: 6- rs12363125; 7- rs2283265; 8- rs2242592; 9- rs1554929; 10- rs2234689

^*b *^***TH***: 1- rs6356; 2- rs2070762

^*c *^Under-represented in patients in comparison with control subjects

Multiple-marker analysis of the two SNPs (rs6356 and rs2070762) in *TH *showed a different overall distribution between cases and controls (best adjusted P-value = 0.015, Table 2), due to an over-representation the A-C allelic combination in cases (P = 0.037, OR = 1.34, 95%CI = 0.94-1.90), and G-T in controls (P = 0.00573, OR = 1.47, 95%IC = 1.11-1.94; Table 3). However, individual haplotype assignation did not identify differences in the frequency of risk haplotype carriers between cases and controls. Moreover, the analysis of rs6356-rs2070762 haplotype distributions in cases and controls of population 2 found no evidence of association with migraine (table 3).

Finally, we aimed to determine whether those variants nominally associated with the disease phenotype in population 1 after the single-marker analysis, could be replicated in population 2, especially rs2283265 in the *DRD2 *gene and rs2070762 in the *TH *gene, which maintained significance after FDR correction in the initial analysis. The comparison of genotype and allele frequencies between cases - either the whole group of migraineurs, or MO or MA subgroups- and controls did not reveal significant differences for rs2283265 (codominant genotypes P = 0.62 and alleles P = 0.36), rs2070762 (codominant genotypes P = 0.44 and alleles P = 0.22) nor for any other SNP (table 1).

## Discussion

We performed a two-stage case-control association study of eight dopamine-related genes in the Spanish population. In order to capture the common haplotype variation of these genes in the European population, we selected haplotype-tagging SNPs which covered each gene and its flanking regions. In population 1, a five-marker risk haplotype in the *DRD2 *gene and a single variant in the *TH *gene were found to be associated with migraine, and both remained significant after applying correction for multiple comparisons. In the initial single-marker analysis, pointing at five genes including the two above, no SNP withstood the Bonferroni correction. However, it is well known that this correction is often over-conservative as it assumes independence of all the tests performed, whereas many SNPs within the genes studied, although not in strong LD, are not independent. When markers found associated in population 1 were analyzed in the follow-up population, the results could not be replicated. As special attention was paid to rule out the existence of stratification and both populations were comparable in terms of size, gender distribution, ethnicity (Caucasians), geographical origin (Spain) and diagnostic criteria, failure to replicate the results suggests that the associations identified in population 1 may be spurious and that the genes analyzed here would not be involved in migraine susceptibility. However, these findings should be taken with caution, as the genetic coverage of some of the studied genes is not optimal for several reasons: First, SNPs with low frequencies, which would require very large sample sizes to produce significant results, were not selected. Second, some SNPs within the studied genes, for which no LD data were available in the HapMap database, were not included. And third, SNPlex design constraints and low genotyping call rates of some specific SNPs forced additional exclusions that left the *DRD4 *gene out of the study. Of note, the same Spanish cohort analyzed in the present work was previously scrutinized by us to detect association of MA or MO with genes related with serotonin neurotransmission [10]. Among the three genes that displayed significant association, two belong to the dopamine metabolic pathway: *MAOA*, found to be associated with MO, and *DDC*, which was associated with MA. However, these findings still await replication.

A number of association studies have focused on dopamine-related genes. The first susceptibility polymorphism identified in this system was the *Nco*I variant in the *DRD2 *gene (rs6275), with an over-representation of the C allele in MA [24]. Subsequent studies failed to replicate this association [25-28] or that with other *DRD2 *polymorphisms [27,29]. It is worth mentioning, however, that a (TG)n repeat variant in *DRD2 *was found associated with yawning and nausea in a small subgroup of migraine patients [30]. We analyzed *DRD2 *rs2242592, in strong LD with rs6275, that belonged to a risk haplotype identified in population 1 but not confirmed in population 2. Subsequent studies found association between migraine phenotypes and polymorphisms in *DRD4 *[31,32] and *DBH *[9,25,33,34], although negative associations have also been described [9,30,31,33]. No associations have been identified in any of the polymorphisms analyzed in genes *DRD1*, *DRD3*, *DRD5 *or *COMT *[9,27,30,35-38]. The genetic marker set selected in the present analysis is in many respects not comparable with the polymorphisms analyzed in the previous studies. However, our results agree with previous negative findings in *DRD1*, *DRD3 *and one polymorphism in *DBH*.

A recent study carried in two German populations [9], analyzed the contribution of the nine dopamine-related genes we have examined, plus *DDC*, to MA susceptibility. In that study, MA was associated with three SNPs, *SLC6A3 *rs40184, *DRD2 *rs7131056 and *DBH *rs2097629. Overall, they analyzed 43 SNPs belonging to the nine genes studied in our work; of them, 23 SNPs are coincident or in strong LD (r^2 ^> 0.85) with the ones we analyzed. The remaining 20 markers were not included in our analysis because their MAFs values were under the selected cut off (n = 13), lacked genotyping in the HapMap sample (n = 3), had SNPlex design constraints (n = 2) or failed in the genotyping step (n = 2). Conversely, our study included 27 SNPs with MAF>0.15 that were not analyzed by Todt et al. Three out of five nominal associations identified in our population 1, not replicated in population 2, also showed P-values > 0.05 in the first German population, thus reinforcing the likelihood of a spurious association in our population 1. Our study did not reveal association with rs7131056 in *DRD2 *or rs40184 in *SLC6A3 *at variance with the German study, while rs2097629 in *DBH *was not included in our study because of design constraints. In addition to differences in the respective SNP sets, our samples were composed of both MO and MA patients, and therefore a comparison of our results with those of Todt et al. is not altogether straightforward. Also, our analytical design set that the two population samples could only be grouped for analysis in case nominal associations were found in both populations 1 and 2, while in the German study their two samples were analyzed as a single group for all SNPs within the three genes that showed nominal association in only one population. This strategy produced significant associations despite lack of replication in their follow-up sample. Future studies combining both marker sets might help to reconcile these apparently discordant findings.

Much evidence points to dopamine hypersensitivity in migraineurs, particularly those displaying the premonitory symptoms of yawning or nausea. In our study, such specific symptoms could not be analyzed, since they were not available in the whole sample. To our knowledge, no well-powered association study has addressed the relationship between endophenotypes based on dopaminergic symptoms and genetic susceptibility using a pathway-based approach. Alternatively, latent class analysis of migraine symptoms, as used to enhance clinical homogeneity in genetic linkage analysis [39,40], might define migraine phenotypes, not necessarily related to ICHD-II migraine subtype diagnoses, and thus uncover specific genetic susceptibility factors.

## Conclusion

In summary, our results do not support the involvement of a set of dopamine-related genes in the genetic vulnerability to migraine in the Spanish population, albeit a previous association study in the same cohort identified *DDC *and *MAOA *as potential susceptibility genes [10]. Further studies in larger samples or family-based sets may help to clarify the contribution of dopamine-related genes to migraine genetic background.

## Competing interests

The authors declare that they have no competing interests.

## Authors' contributions

RC carried out the genotyping, analyzed the data and drafted the manuscript. MC, EC-L and MJS participated in genotyping. BC and AM designed the study and had the primary responsibility for writing the manuscript. MR helped in study design and supervised all the statistical analysis. JP, SB, MJS and AM were responsible for selecting and evaluating the patients in the respective centers. EC-L, MJS and RC participated in control recruitment. All authors have read and approved the final manuscript.

## Pre-publication history

The pre-publication history for this paper can be accessed here:



## Supplementary Material

Additional file 1 **Supplementary table S1**. Description of the SNPs initially selected for the SNPlex analysis within 9 dopaminergic candidate genes for migraine. Click here for file

Additional file 2 **Supplementary table S2**. Hardy-Weinberg equilibrium, minimal allele frequency (MAF) and nominal P-values observed when genotype and allele frequencies of 50 SNPs within 8 candidate genes were considered in 263 migraine cases and 274 unrelated migraine-free controls. Click here for file

Additional file 3 **Supplementary table S3**. Assessment of population stratification using 45 unlinked anonymous SNPs and the Structure v2.1 software. Click here for file
