# Supplementary material for: Two-stage case-control association study of dopamine-related genes and migraine
Source: BMC Med Genet. 2009 Sep 21;10:95. doi: 10.1186/1471-2350-10-95 (PMC2758864; doi:10.1186/1471-2350-10-95)
Supplement: Additional file 1 — Supplementary table S1. Description of the SNPs initially selected for the SNPlex analysis within 9 dopaminergic candidate genes for migraine. [file 1471-2350-10-95-S1.DOC]

**Supplementary table 1**. Description of the SNPlex assay within 9 dopamine-related candidate genes for migraine.

| **Gene** | **Contig reference** | **Location** | **Length (kp)** | **N of exons** | **SNPs** | **Tag SNPs** | **SNP ID** | **Location** | **Exclusion criteria** | **Gene Coverage**** | **Other SNPs within the BIN** |
| --- | --- | --- | --- | --- | --- | --- | --- | --- | --- | --- | --- |
| ***DRD1*** | NM_00794 | Chr 5q35.1 | 3.127 | 2 | 11 | 8 | rs4867798 | Exon 2 | Failed | 0.87 | - |
|  |  |  |  |  |  |  | rs251937 | 3’ |  |  | rs703748 |
|  |  |  |  |  |  |  | rs11749676 | 3’ |  |  | rs686, rs265978 |
|  |  |  |  |  |  |  | rs835540 | 3’ |  |  | - |
|  |  |  |  |  |  |  | rs835616 | 3’ |  |  | - |
|  |  |  |  |  |  |  | rs835541 | 3’ |  |  | - |
|  |  |  |  |  |  |  | rs863126 | 3’ |  |  | - |
|  |  |  |  |  |  |  | rs265977 | 3’ |  |  | - |
| ***DRD2*** | NM_000795 | Chr 11q23 | 65.56 | 8 | 35 | 10 | rs4630328 | Intron 1 |  | 1 | rs17601612 |
|  |  |  |  |  |  |  | rs7131056 | Intron 1 |  |  | - |
|  |  |  |  |  |  |  | rs4245146 | Intron 1 |  |  | rs4245147, rs4274224 |
|  |  |  |  |  |  |  | rs17529477 | Intron 1 |  |  | - |
|  |  |  |  |  |  |  | rs2002453 | Intron 1 |  |  | rs1076562, rs2005313, rs2245805, rs4586205, rs4648318 |
|  |  |  |  |  |  |  | rs12363125 | Intron 2 |  |  | rs1076563, rs12364051, rs12800853, rs2587548, rs2734833, rs2734837, rs2734838, rs2734839, rs4587762, rs4938017, rs7122246, rs7131440 |
|  |  |  |  |  |  |  | rs2283265 | Intron 5 |  |  | - |
|  |  |  |  |  |  |  | rs2242592 | 3’ |  |  | rs2734841, rs2734842, rs6275, rs6279 |
|  |  |  |  |  |  |  | rs1554929 | 3’ |  |  | rs10891549 |
|  |  |  |  |  |  |  | rs2234689 | 3’ |  |  | - |
| ***DRD3*** | NM_000796 | Chr 3q13.3 | 50.34 | 7 | 23 | 11 | rs9825563 | 5’ |  | 1 | - |
|  |  |  |  |  |  |  | rs1800828 | Intron 1 | LD* |  | - |
|  |  |  |  |  |  |  | rs6280 | Exon 2 |  |  | rs324026, rs7638876 |
|  |  |  |  |  |  |  | rs10934256 | Intron 2 |  |  | rs7633291 |
|  |  |  |  |  |  |  | rs167770 | Intron 2 | LD* |  | rs226082,rs324030,rs7625282,rs324029, rs324022, rs11721264 |
|  |  |  |  |  |  |  | rs167771 | Intron 3 |  |  | - |
|  |  |  |  |  |  |  | rs324035 | Intron 3 | LD* |  | - |
|  |  |  |  |  |  |  | rs9880168 | Intron 3 |  |  | - |
|  |  |  |  |  |  |  | rs2134655 | Intron 5 |  |  | - |
|  |  |  |  |  |  |  | rs3732790 | 3’ |  |  | rs963468, rs3773679 |
|  |  |  |  |  |  |  | rs2399496 | 3’ | Failed |  | rs9817063 |
| ***DRD4*** | NM_000797 | Chr 11p15.5 | 3.399 | 4 | 4 | 2 | rs3758653 | 5’ | SNPlex design | 0 | - |
|  |  |  |  |  |  |  | rs936465 | 3’ | Failed |  | rs4331145, rs11246226 |
| ***DRD5*** | NM_000798 | Chr 4p16.1 | 2.03 | 1 | 3 | 2 | rs2227850 | Exon 1 | Monomorphic | 1 |  |
|  |  |  |  |  |  |  | rs10033951 | 3’ |  |  | rs2867383 |
| ***COMT*** | NM_000754 | Chr22q11.21 | 27.22 | 6 | 14 | 10 | rs2020917 | 5’ |  | 0.8 | rs737866 |
|  |  |  |  |  |  |  | rs933271 | Intron 1 |  |  | rs174675 |
|  |  |  |  |  |  |  | rs1544325 | Intron 1 |  |  | rs5993883 |
|  |  |  |  |  |  |  | rs740603 | Intron 1 |  |  | - |
|  |  |  |  |  |  |  | rs740601 | Intron 3 |  |  | rs2239393 |
|  |  |  |  |  |  |  | rs4680 | Exon 4 | Failed |  | - |
|  |  |  |  |  |  |  | rs4646316 | Intron 5 |  |  | - |
|  |  |  |  |  |  |  | rs174696 | Intron 5 | SNPlex design |  | - |
|  |  |  |  |  |  |  | rs165774 | Intron 5 |  |  | - |
|  |  |  |  |  |  |  | rs9332377 | Intron 5 |  |  | - |
| ***DBH*** | NM_000787 | Chr 9q34 | 22.98 | 12 | 22 | 14 | rs2007153 | Intron 1 |  | 0.86 | - |
|  |  |  |  |  |  |  | rs2797851 | Intron 1 |  |  | rs2519155, rs279785, rs2797849 |
|  |  |  |  |  |  |  | rs1548364 | Intron 3 |  |  | rs1611122, rs1611125, rs2873804 |
|  |  |  |  |  |  |  | rs2797855 | Intron 5 |  |  | rs1611123 |
|  |  |  |  |  |  |  | rs1541332 | Intron 5 |  |  | - |
|  |  |  |  |  |  |  | rs2519154 | Intron 5 |  |  | - |
|  |  |  |  |  |  |  | rs2797853 | Intron 5 |  |  | - |
|  |  |  |  |  |  |  | rs6479643 | Intron 6 |  |  | - |
|  |  |  |  |  |  |  | rs77905 | Exon 9 |  |  | - |
|  |  |  |  |  |  |  | rs2097628 | Intron 9 | SNPlex design |  | rs2097629 |
|  |  |  |  |  |  |  | rs2073833 | Intron 9 |  |  | - |
|  |  |  |  |  |  |  | rs1611131 | Intron 10 |  |  | - |
|  |  |  |  |  |  |  | rs129883 | 3’ | Failed |  | - |
|  |  |  |  |  |  |  | rs129915 | 3’ | SNPlex design |  | - |
| ***SLC6A3*** | NM_001044 | Chr 5p15.3 | 52.63 | 15 | 19 | 9 | rs2617605 | Intron 2 | Failed | 0.45 | - |
|  |  |  |  |  |  |  | rs460700 | Intron 4 | Failed |  | rs464061, rs456082, rs409588, rs458860, rs464528, rs463379, rs456774, rs460000 |
|  |  |  |  |  |  |  | rs37020 | Intron 6 |  |  | rs464049, rs458334 |
|  |  |  |  |  |  |  | rs13161905 | Intron 6 |  |  | - |
|  |  |  |  |  |  |  | rs27048 | Intron 8 |  |  | - |
|  |  |  |  |  |  |  | rs6347 | Exon 9 | Failed |  | - |
|  |  |  |  |  |  |  | rs11133767 | Intron 13 | Failed |  | - |
|  |  |  |  |  |  |  | rs40184 | Intron 14 |  |  | - |
|  |  |  |  |  |  |  | rs2975292 | 3’ | SNPlex design |  | - |
| ***TH*** | NM_199292 | Chr 11p15.5 | 7.876 | 14 | 4 | 3 | rs10770140 | 5’ | Failed | 0.66 | rs10770141 |
|  |  |  |  |  |  |  | rs6356 | Exon 2 |  |  | - |
|  |  |  |  |  |  |  | rs2070762 | Intron 13 |  |  | - |

*LD with other SNPs within the gene in the sample of 274 controls

** Analyzed tagSNPs/total tagSNPs within the gene

LD: Linkage Disequilibrium

HWE: Hardy-Weinberg equilibrium

N: Number
