# Supplementary material for: Two-stage case-control association study of dopamine-related genes and migraine
Source: BMC Med Genet. 2009 Sep 21;10:95. doi: 10.1186/1471-2350-10-95 (PMC2758864; doi:10.1186/1471-2350-10-95)
Supplement: Additional file 2 — Supplementary table S2. Hardy-Weinberg equilibrium, minimal allele frequency (MAF) and nominal P-values observed when genotype and allele frequencies of 50 SNPs within 8 candidate genes were considered in 263 migraine cases and 274 unrelated migraine-free controls. [file 1471-2350-10-95-S2.DOC]

**Supplementary table 2**. Hardy-Weinberg equilibrium, minimal allele frequency (MAF) and single SNP association results of 263 migraine cases and 274 unrelated migraine-free controls.

| ***Gene*** | **SNP** | **Hardy-Weinberg equilibrium in controls (P)** | **MAF** | **Codominant genotype**  **P-value** | **Allelic**  **P-value** |
| --- | --- | --- | --- | --- | --- |
| ***DRD1*** | rs251937 | 0.671284 | 0.32 | 0.07798 | 0.02611 |
|  | rs11749676 | 0.420685 | 0.344 | 0.46106 | 0.48870 |
|  | rs835540 | 0.654498 | 0.289 | 0.33643 | 0.14980 |
|  | rs835616 | 0.875605 | 0.281 | 0.36914 | 0.37896 |
|  | rs835541 | 0.320660 | 0.473 | 0.36422 | 0.64986 |
|  | rs863126 | 0.795432 | 0.372 | 0.28403 | 0.11764 |
|  | rs265977 | 0.086653 | 0.173 | 0.08033 | 0.50514 |
| ***DRD2*** | rs4630328 | 0.444532 | 0.4 | 0.09492 | 0.13767 |
|  | rs7131056 | 0.708333 | 0.41 | 0.47641 | 0.23569 |
|  | rs4245146 | 0.807639 | 0.5 | 0.12300 | 0.22992 |
|  | rs17529477 | 0.895922 | 0.364 | 0.41791 | 0.34927 |
|  | rs2002453 | 1.000000 | 0.198 | 0.82138 | 0.93222 |
|  | rs12363125 | 0.423392 | 0.353 | 0.03036 | 0.04012 |
|  | rs2283265 | 1.000000 | 0.149 | 0.00848 | 0.00299 |
|  | rs2242592 | 1.000000 | 0.253 | 0.28630 | 0.12885 |
|  | rs1554929 | 0.525792 | 0.406 | 0.60235 | 0.80363 |
|  | rs2234689 | 0.380185 | 0.167 | 0.44690 | 0.49077 |
| ***DRD3*** | rs9825563 | 0.776063 | 0.31 | 0.50827 | 0.24033 |
|  | rs6280 | 1.000000 | 0.33 | 0.36645 | 0.19298 |
|  | rs10934256 | 0.483116 | 0.221 | 0.39115 | 0.16370 |
|  | rs167771 | 0.669495 | 0.172 | 0.91093 | 0.67221 |
|  | rs9880168 | 0.094178 | 0.123 | 0.87076 | 0.75954 |
|  | rs2134655 | 1.000000 | 0.249 | 0.58128 | 0.30065 |
|  | rs3732790 | 0.075775 | 0.4 | 0.01305 | 0.01688 |
| ***DRD5*** | rs10033951 | 0.258767 | 0.324 | 0.37453 | 0.48171 |
| ***COMT*** | rs2020917 | 0.048782 | 0.32 | 0.44521 | 0.46923 |
|  | rs933271 | 1.000000 | 0.334 | 0.23916 | 0.21630 |
|  | rs1544325 | 0.890810 | 0.339 | 0.05330 | 0.05960 |
|  | rs740603 | 0.175927 | 0.362 | 0.14884 | 0.51378 |
|  | rs740601 | 0.901975 | 0.463 | 0.84297 | 0.56569 |
|  | rs4646316 | 0.207067 | 0.261 | 0.35996 | 0.17157 |
|  | rs165774 | 0.358586 | 0.279 | 0.71598 | 0.87129 |
|  | rs9332377 | 0.833804 | 0.18 | 0.96863 | 0.85606 |
| ***DBH*** | rs2007153 | 0.891207 | 0.345 | 0.05921 | 0.63654 |
|  | rs2797851 | 0.790209 | 0.361 | 0.79766 | 0.97669 |
|  | rs1548364 | 0.619964 | 0.45 | 0.59401 | 0.32857 |
|  | rs2797855 | 0.369534 | 0.418 | 0.81602 | 0.74235 |
|  | rs1541332 | 0.712722 | 0.456 | 0.57183 | 0.30985 |
|  | rs2519154 | 0.374847 | 0.413 | 0.25506 | 0.64736 |
|  | rs2797853 | 0.181805 | 0.379 | 0.68524 | 0.55925 |
|  | rs6479643 | 0.245367 | 0.388 | 0.09802 | 0.48581 |
|  | rs77905 | 0.902118 | 0.481 | 0.77548 | 0.98515 |
|  | rs2073833 | 0.112572 | 0.398 | 0.06263 | 0.84515 |
|  | rs1611131 | 0.285523 | 0.285 | 0.04002 | 0.49551 |
| ***SLC6A3*** | rs37020 | 0.797777 | 0.395 | 0.94916 | 0.91921 |
|  | rs13161905 | 0.614387 | 0.431 | 0.87783 | 0.95200 |
|  | rs27048 | 0.236727 | 0.383 | 0.69879 | 0.55986 |
|  | rs40184 | 0.219444 | 0.468 | 0.15882 | 0.89022 |
| ***TH*** | rs6356 | 0.693085 | 0.376 | 0.68753 | 0.44769 |
|  | rs2070762 | 1.000000 | 0.431 | 0.01303 | 0.01109 |
