# Supplementary material for: Two-stage case-control association study of dopamine-related genes and migraine
Source: BMC Med Genet. 2009 Sep 21;10:95. doi: 10.1186/1471-2350-10-95 (PMC2758864; doi:10.1186/1471-2350-10-95)
Supplement: Additional file 3 — Supplementary table S3. Assessment of population stratification using 45 unlinked anonymous SNPs and the Structure v2.1 software. [file 1471-2350-10-95-S3.DOC]

**Supplementary table 3.** Assessment of population stratification using 45 unlinked anonymous SNPs and the Structure v2.1 software

| **Number of subpopulations (K)** | **LnP(D)^a^** | **SD[LnP(D) ^b^** | **Posterior probability of K ^c^** |
| --- | --- | --- | --- |
| Population 1 (263 cases and 274 controls) | | | |
| 1 | -28956.9 | 0.51 | 1 |
| 2 | -29255.5 | 244.88 | 2.04e-130 |
| 3 | -29440.1 | 271.94 | 3.68e-306 |
| 4 | -29276.7 | 299.11 | 1.30e-139 |
| 5 | -29199.4 | 247.47 | 4.64e-106 |
| Population 2 (259 cases and 287 controls) | | | |
| 1 | -31381.3 | 0.228 | 1 |
| 2 | -31411.7 | 32.73 | 5.9e-14 |
| 3 | -31457.4 | 53.49 | 3.68e-306 |
| 4 | -31430.4 | 19.39 | 4.47e-22 |
| 5 | -31445.4 | 39.49 | 1.37e-28 |

**^a^** Ln of the probability of the data, calculated as an average of five independent iterations, with burning period and number of MCMC repeats set to 100,000. An admixture ancestry model with correlated allele frequencies (λ=1) was assumed.

**^b^** Standard Deviation of the five LnP(D) iterations.

**^c^** Calculated using Bayes’ rule.
